# Soil carbon dioxide venting through rice roots

**DOI:** 10.1111/pce.13638

**Published:** 2019-08-19

**Authors:** Guy J.D. Kirk, Andrea Boghi, Marie‐Cecile Affholder, Samuel D. Keyes, James Heppell, Tiina Roose

**Affiliations:** ^1^ School of Water, Energy and Environment Cranfield University Cranfield UK; ^2^ Faculty of Engineering and Environment University of Southampton Southampton UK

**Keywords:** biological models, biological transport, X‐ray computed tomography

## Abstract

The growth of rice in submerged soils depends on its ability to form continuous gas channels—aerenchyma—through which oxygen (O_2_) diffuses from the shoots to aerate the roots. Less well understood is the extent to which aerenchyma permits venting of respiratory carbon dioxide (CO_2_) in the opposite direction. Large, potentially toxic concentrations of dissolved CO_2_ develop in submerged rice soils. We show using X‐ray computed tomography and image‐based mathematical modelling that CO_2_ venting through rice roots is far greater than thought hitherto. We found rates of venting equivalent to a third of the daily CO_2_ fixation in photosynthesis. Without this venting through the roots, the concentrations of CO_2_ and associated bicarbonate (HCO_3_^−^) in root cells would have been well above levels known to be toxic to roots. Removal of CO_2_ and hence carbonic acid (H_2_CO_3_) from the soil was sufficient to increase the pH in the rhizosphere close to the roots by 0.7 units, which is sufficient to solubilize or immobilize various nutrients and toxicants. A sensitivity analysis of the model showed that such changes are expected for a wide range of plant and soil conditions.

## Abstract 2

Large, potentially toxic concentrations of dissolved CO_2_ accumulate in submerged paddy soils because CO_2_ from plant and soil respiration escapes only very slowly. We found, using X‐ray computed tomography and image‐based mathematical modelling, venting of soil CO_2_ through rice roots at rates equivalent to a third of the daily CO_2_ fixation in photosynthesis. Without this venting, the concentrations of CO_2_ and associated bicarbonate in root cells would have been well above levels known to be toxic to roots. Removal of soil CO_2_ and hence carbonic acid will also affect the solubility and hence plant uptake of various nutrients and toxicants in the rhizosphere.

## 1 INTRODUCTION

**Figure 1:**
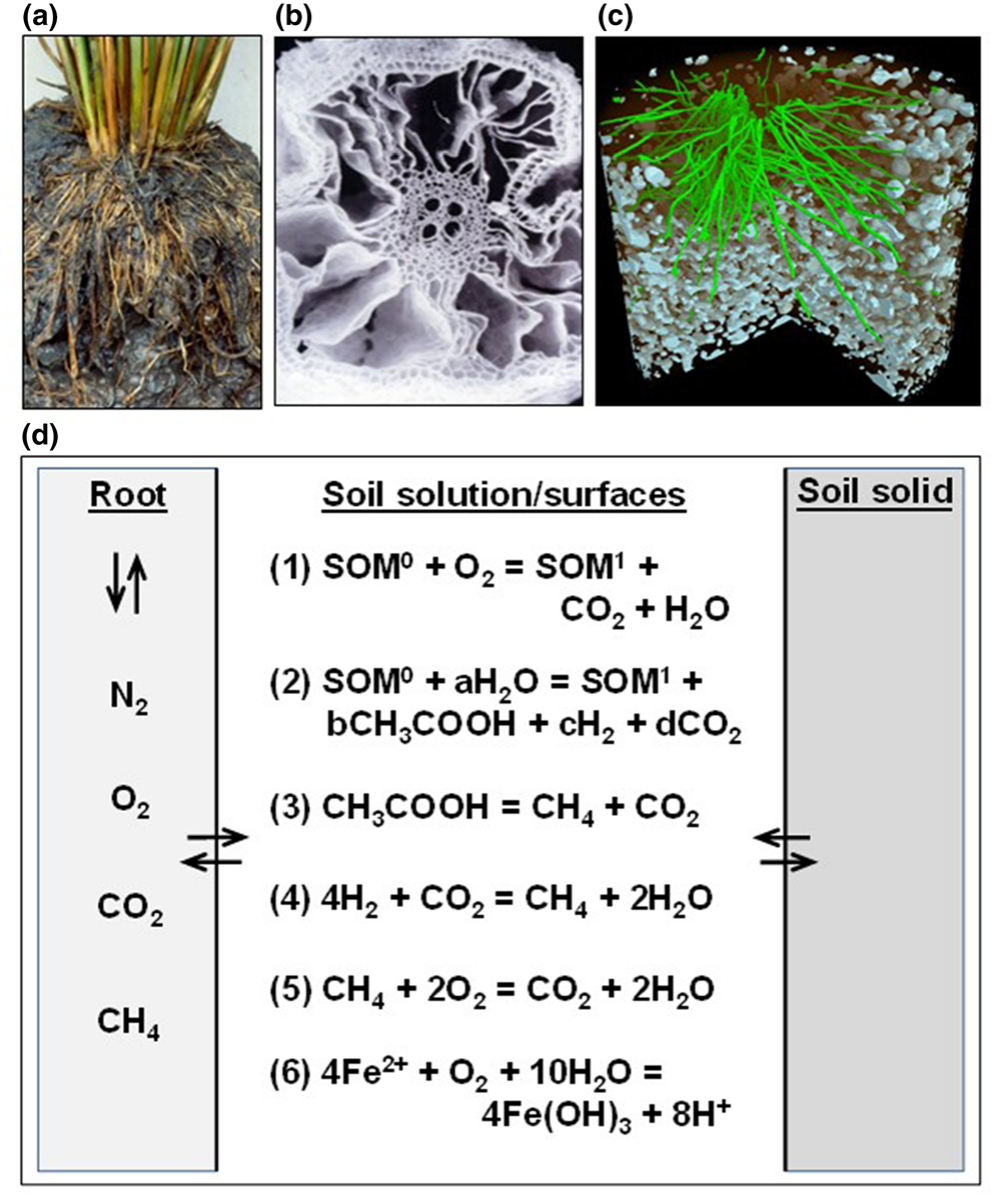
Gas formation and venting through rice roots in paddy soil. (a) Cross section showing roots and water‐saturated, anaerobic soil. (b) Root aerenchyma. (c) Cut‐away X‐ray computed tomography image of roots (green) and soil gas bubbles (white). (d) Gas generating and consuming processes in the soil (after inorganic oxidants have been exhausted): (1) aerobic decomposition of soil organic matter (SOM) in the rhizosphere, (2) anaerobic decomposition of SOM in the soil bulk (a–d are coefficients), (3) CH_4_ production from acetate, (4) CH_4_ production from H_2_, (5) CH_4_ oxidation, and (6) Fe (II) oxidation. Gas bubbles become entrapped under soil particles, but there is no continuous gas phase through the soil [Colour figure can be viewed at http://wileyonlinelibrary.com]

Large dissolved CO_2_ concentrations develop in submerged rice soils (equivalent partial pressures 5–70 kPa—Greenway, Armstrong, & Colmer, 2006; Kirk, 2004; Ponnamperuma, 1972) because CO_2_ formed in root and soil respiration escapes only slowly by diffusion through the water‐filled soil pores. Carbon dioxide is produced in anaerobic respiration in the soil bulk and in aerobic respiration in the rhizosphere fuelled by O_2_ and organic substrates released from the roots (Figure 1). There is therefore a large CO_2_ gradient between the soil and the aerenchyma inside the root. Hence, CO_2_ will enter the roots by diffusion and mass flow in the transpiration stream and be vented to the shoots and atmosphere by diffusion through the aerenchyma (Higuchi, Yoda, & Tensho, 1984). There has been much research on this pathway for CH_4_ emission from ricefields (Butterbach‐Bahl, Papen, & Rennenberg, 1997; Nouchi, Mariko, & Aoki, 1990; Schütz, Seiler, & Conrad, 1989; Wang, Akiyama, Yagi, & Yan, 2018), but CO_2_—which is >20 times less volatile than CH_4_—has received little attention. High CO_2_ concentrations and associated HCO_3_^−^ can be toxic to root cells, and therefore, some degree of venting is necessary for healthy growth (Greenway et al., 2006). Also, removal of dissolved CO_2_ will tend to increase the pH of the rhizosphere soil, with consequences for the ricefield biogeochemistry (Affholder, Weiss, Wissuwa, Johnson‐Beebout, & Kirk, 2017; Begg, Kirk, MacKenzie, & Neue, 1994; Kirk & Bajita, 1995). Two further processes affect the chemistry of the rice rhizosphere: oxidation of inorganic reductants, such as ferrous iron, by O_2_ from the roots and associated generation of H^+^, and release of H^+^ from the roots to balance excess intake of cations (particularly NH_4_^+^) over anions (Kirk, 2004). These inputs of H^+^ will tend to offset H^+^ consumption in venting of dissolved CO_2_ from the soil and the resulting changes in carbonate equilibria.

Investigating such processes is challenging given the sensitivity of gas fluxes to measurement conditions. A key problem is how to separate the fluxes of soil‐derived CO_2_ from those of root‐ and shoot‐derived CO_2_. This might be done, for example, with isotopically labelled carbon sources, if it were possible to ensure uniform labelling and complete separation of the plant and soil sources. In this study, we avoided these difficulties by directly imaging and quantifying profiles of gas depletion around rice roots growing in submerged soil using X‐ray computed tomography (CT) and mathematical modelling.

In brief, we grew initially 4‐week‐old rice seedlings in a submerged, anaerobic rice soil contained in glass pots, and, after 4 weeks, scanned the pots using X‐ray CT imaging to measure the spatial distribution of roots and gas bubbles entrapped in the soil (Figure 1c). The image analysis showed prominent and abundant gas bubbles in the soil bulk, but no or very few bubbles in the soil close to roots, and there was a clear relation between the absence of gas bubbles and high root density, as well as an increasing concentration of bubbles with depth through the soil. Analysis of the bubbles showed they were approximately 40% CO_2_ by volume and 60% CH_4_. We developed a mathematical model to account for these observations on the basis of the following picture of events.

If the soil solution becomes supersaturated with CO_2_ or CH_4_, or other volatile products of respiration, gas bubbles will form and tend to become entrapped beneath soil particles. If the bubbles become sufficiently large, or if the soil is agitated by some mechanical disturbance, then the bubbles will rise to the surface by “ebullition.” At steady state (which is typically reached within a few weeks of the soil being submerged—Kirk, 2004; Ponnamperuma, 1972), the volume of bubbles and their composition, as well as the concentrations of dissolved gases in equilibrium with them, will depend on the rates of production versus loss by ebullition and diffusion and venting through the roots. We fitted the model, on the basis of this outline, to the X‐ray CT images of roots and gas bubbles. Thereby, we obtained values of the model parameters and the proportions of CO_2_ and CH_4_ generated in and leaving the soil via the various pathways. The details follow.

## 2 MATERIALS AND METHODS

### 2.1 Model development

We describe the steady‐state transport of each dissolved gas through the soil by the following continuity equation:

(1) $$\nabla \cdot \left(D_{i}\nabla C_{Li}-{\mathrm{vC}}_{Li}\right)+S_{i}-E_{i}-R_{i}=0,$$

where *C*_L*i*_ is the concentration of dissolved gas *i*, *D*_*i*_ is its diffusion coefficient through the soil solution, *v* is the water flux into roots, *S*_*i*_ is the rate of gas production, *E*_*i*_ is the rate of ebullition, and *R*_*i*_ is the rate of root‐mediated efflux. There is an equation of this form each for dissolved CO_2_, CH_4_, and N_2_, which enters the soil by diffusion from the atmosphere and roots. For CO_2_, *C*_L*i*_ is adjusted for the concentration of dissolved CO_2_ plus the concentration of HCO_3_^−^ in equilibrium with it (CO_3_^2−^ is unimportant at the near neutral pH of most submerged soils).

In Equation (1), the diffusion coefficient, *D*_*i*_ = *D*_L*i*_ *θ*_L_ *f*_L_ where *D*_L*i*_ is the diffusion coefficient in free solution, *θ*_L_ is the soil volumetric water content, and *f*_L_ is a tortuosity factor (Kirk, 2004). The volumetric gas content, *θ*_G_ (from which *θ*_L_ = *θ* − *θ*_G_ where *θ* is the total porosity) is proportional to the sum of the partial pressures of the volatile solutes, $\sum P_{i}=P_{{\mathrm{CO}}_{2}}+P_{{\mathrm{CH}}_{4}}+P_{N_{2}}+P_{H_{2}O}$ ( $P_{H_{2}O}$ is the saturating pressure of H_2_O):

(2) $$\theta_{G}=K_{\theta }\sum P_{i},$$

where *K*_*θ*_ is a constant that is characteristic of the submerged, puddled soil. From the gas law: *P*_*i*_ = *RTC*_G*i*_ where *C*_G*i*_ is the concentration of gas *i* in the soil gases. From Henry's law: *C*_G*i*_ = *C*_L*i*_/*K*_H*i*_ where *K*_H*i*_ is the dimensionless Henry's law constant for gas *i*.

We specify the following relations for *S*_*i*_, *E*_*i*_, and *R*_*i*_. For *S*_*i*_, at steady state, CO_2_ production from soil carbon is constant with depth and time, equal to $S_{{\mathrm{CO}}_{2},0}$, and production from root‐derived carbon is proportional to the root length density, *L*_V_ (root length per unit soil volume), that is,

(3) $$S_{{\mathrm{CO}}_{2}}=S_{{\mathrm{CO}}_{2},0}+k_{V}L_{V},$$

where *k*_V_ is a proportionality constant. At steady state, the ratio of CH_4_ production to CO_2_ production is also constant (Kirk, 2004):

(4) $$S_{{\mathrm{CH}}_{4}}=\alpha_{{\mathrm{CH}}_{4}}S_{{\mathrm{CO}}_{2}}.$$

For *E*_*i*_, the rate of ebullition is a function of the volume of the gas bubbles: As bubbles grow, they become more buoyant and so are more easily displaced. Hence, taking total gas volume to represent bubble volume:

(5) $$E_{i}=k_{E}\theta_{G}C_{Gi},$$

where *k*_E_ is a rate constant that depends on the physical properties of the soil. For *R*_*i*_, root‐mediated efflux from the soil occurs by degassing of dissolved CO_2_ and CH_4_ into the root aerenchyma and diffusion through the aerenchyma to the atmosphere (Beckett, Armstrong, Justin, & Armstrong, 1988). We represent this as

(6) $$R_{i}=k_{T}L_{V}D_{Gi}\left(C_{Gi}-C_{Gi0}\right),$$

where *k*_T_ is a root gas transmissivity, *D*_G*i*_ is the diffusion coefficient of gas *i* in air, *C*_G*i*_ is the gas concentration along the profile, and C_G*i*0_ is the gas concentration at *z* = 0. The root gas transmissivity accounts for all factors limiting CO_2_ transfer from the soil solution at the root surface to the aerenchyma at the base of the roots at *z* = 0, including the gas permeability of the root wall and epidermis, and the root porosity.

We solved Equations (1)–(6) subject to *C*_L*i*_ being constant at the soil–floodwater boundary and there being no flux of gases across the lower boundary. We fitted the model to the observed profiles of gas content by optimizing the values of *k*_V_, *k*_E_, and *k*_T_; all the other parameters were derived independently, and a single set of values was fitted for all replicates and both planting densities (Section 2.4 and Table 1).

**Table 1:** Standard parameter values

Symbol	Definition	Standard value	Comments
*L*	Soil depth	1.7 dm	Set by experimental conditions
*θ*	Soil porosity	0.69	Measured
*f*_L_	Soil liquid diffusion impedance factor	0.35	Based on Kirk, Solivas, & Alberto (2003) for similar soils
*v*	Water flux into roots	0 dm s^−1^	At *v* = 10^−7^ dm s^−1^, which is a typical value (Kirk, 2004), the additional CO_2_ flux into the roots (=*vC*_L_) is <2% greater. We therefore use *v* = 0 for simplicity.
pH	Soil pH	7.0	Measured
[H_2_CO_3_^*^]_0_ + [HCO_3_^−^]_0_	H_2_CO_3_^*^ + HCO_3_^−^ concentration at *z* = 0	1 mM	Measured
[H_2_CO_3_^*^]_*i*_ + [HCO_3_^−^]_*i*_	H_2_CO_3_^*^ + HCO_3_^−^ concentration in bulk soil at *t* = 0	40 mM	Measured
[CH_4_]_0_	Dissolved CH_4_ concentration at *z* = 0	2.9 nM	From atmospheric $P_{{\mathrm{CH}}_{4}}$
[N_2_]_0_	Dissolved N_2_ concentration at *z* = 0	0.5 mM	From atmospheric $P_{N_{2}}$
$S_{{\mathrm{CO}}_{2},0}$	CO_2_ production from soil C	9.5 × 10^−8^ mol dm^−3^ s^−1^	Fitted for unplanted soil, such that *C*_L_ in the absence of roots (i.e., *k*_V_ = 0, *k*_T_ = 0) agrees with measured value
$\alpha_{{\mathrm{CH}}_{4}}$	$S_{{\mathrm{CH}}_{4}}/S_{{\mathrm{CO}}_{2}}$	1.0	Set such that $P_{{\mathrm{CH}}_{4}}\approx P_{{\mathrm{CO}}_{2}}$
*K*_*θ*_	Equation (2)	2.3 × 10^−3^	Fitted for unplanted soil
*k*_E_	Rate constant for ebullition	1.0 × 10^−4^ s^−1^	Fitted
*k*_T_	Root gas transmissivity	9.5 × 10^−4^	Fitted
*k*_V_	Constant for decomposition of root‐derived C	9.3 × 10^−13^ mol dm^−1^ s^−1^	Fitted

### 2.2 Experimental methods

We used the same soil, rice genotype, and growth conditions as in Affholder et al. (2017). In brief, 4‐week‐old rice seedlings, grown in nutrient culture, were transplanted into pots of submerged, anaerobic rice soil at either one or four plants per pot planted closely together. After 4 weeks, the pots were scanned using X‐ray CT imaging to measure the spatial distribution of roots and gas bubbles entrapped in the soil (Section 2.3).

The soil was from ricefields at Tiaong, Quezon Province, Philippines. It is a Hydraquent (USDA Soil Taxonomy). Portions of topsoil (0‐ to 30‐cm depth) were air dried and sieved to pass <2 mm. The properties of the sieved soil were 42% clay, 40% silt, pH (aerobic in H_2_O) 8.5, CEC 9.0 cmol_c_ kg^−1^, organic carbon content 73 g kg^−1^, and carbonate content 96 g kg^−1^ (Izquierdo, Impa, Johnson‐Beebout, Weiss, & Kirk, 2016).

Portions (1.2 kg) of the air‐dried soil were mixed with 10 g kg^−1^ of rice straw to stimulate anaerobic reduction processes and then saturated with deionized water and puddled to make a slurry. The slurry was poured into 10‐cm‐internal‐diameter, 21‐cm‐deep, cylindrical, thin‐walled (3‐mm) Perspex pots to a depth of 17 cm. The resulting soil bulk density was 0.81 kg dm^−3^, and the volumetric water content was 0.69. The filled pots were inserted into 12‐cm‐diameter, 21‐cm‐deep glass pots, and the space between the inner and outer pots were filled with further slurry. This arrangement ensured anoxic conditions in the soil in the inner pot, whereas the thin Perspex wall of the pot was completely transparent to X‐rays for imaging after removal from the outer pot. Further deionized water was added to bring the level to the top of the pots, and the water standing in the pots was maintained at this level through the experiment. The soil was allowed to become reduced for 4 weeks at 30°C before transplanting the rice seedlings.

Rice seeds (CV IR55179) were germinated in petri dishes at 30°C in complete darkness for 3 days. The germinated seeds were transferred to a mesh floating on Zn‐free Yoshida nutrient solution (Yoshida, Forno, Cook, & Gomez, 1976) and grown for 4 weeks before being transplanted manually into the prereduced soil in pots. The seedlings were placed with the root crown at approximately 5 cm below the soil–floodwater boundary, as is the practice for growing rice in this soil in the field because of its loose structure and hence weak support for seedlings (Mori et al., 2016). The growth conditions—both before and after transplanting—were 13.5‐hr light (600‐μmol·m^−2^·s^−1^ white light) at 30°C and 10.5‐hr dark at 24°C.

At 4 weeks after transplanting, the inner Perspex pots were removed and the roots and soil in the pots were imaged as described below. The imaging was complete within 24 hr. The aerial plant parts were then separated from roots at the root crown limit. The fresh biomass was measured, and tillers and leaf number were counted. They were then thoroughly washed with UHP water and dried at 70°C for 5 days.

Further pots were set up in the same way but left unplanted to measure gas productions in the bulk soil following flooding. Each pot was fitted with a rhizon solution sampler (Rhizosphere research products, Wageningen, Netherlands) with a 5‐cm porous section and fitted with a Luer lock. The samplers were held vertically in the soil so that the porous section ran from 8.5 to 13.5 cm below the floodwater–soil boundary. At weekly intervals, solution was withdrawn and analysed for dissolved CO_2_ (MI‐720 electrode, Microelectrodes Inc, USA) and pH (MI‐410 combination electrode, Microelectrodes Inc, USA). Redox potential was monitored with a Pt electrode. The composition of gas bubbles accumulated in the soil was monitored by periodically fitting over each pot a 3‐dm^3^ gas‐tight bag fitted with a sampling port and agitating the pots to displace entrapped soil gases into the headspace. Samples of the headspace were withdrawn by syringe and analysed for CO_2_ and CH_4_ by gas chromatography (Cambridge Scientific Instruments 200 Series GC).

We estimate the pH buffer power (i.e., the amount of base required to produce unit increase in pH; *b*_HS_) of the submerged, reduced soil from the results of Affholder et al. (2017) who found with the same rice genotype and growth conditions as here that the pH averaged over the root zone increased by 0.34 pH units due to a net removal of H^+^ as H_2_CO_2_ through the roots of 11.0 mmol kg^−1^ but offset by a net addition of 1.6 mmol H^+^ kg^−1^ from the roots to balance excess intake of cations over anions. On the basis of the soil Fe (II) concentration, the addition of H^+^ in Fe (II) oxidation by the roots was far smaller. Hence, *b*_HS_ = (11.0 − 1.6)/0.32 = 29 mmol·kg^−1^·pH^−1^.

### 2.3 X‐ray CT imaging

Roots and gas bubbles in the pots were imaged using a Custom Nikon/XTEK Hutch X‐ray CT scanner. The field of view was 8 cm in diameter and 5.6 cm in height, with the upper edge approximately at the base of the primary roots, 5 cm below the soil–floodwater boundary. The pots were scanned at 120 kV and 185 uA. A 1‐mm copper filter was used to minimize beam hardening. A total of 3,001 angular projections through 360° were acquired at an exposure of 177 ms, with 32‐frame averaging for each projection. The scan duration was 4.7 hr per sample, and the resulting voxel size was 40 μm (isotropic). Data were reconstructed using a filtered back‐projection algorithm implemented in Nikon CTPro 3D, generating 32‐bit volumes that were subsampled to produce a stack of two‐dimensional eight‐bit Tagged Image File Format files for each scan. A modest beam hardening correction was applied during reconstruction.

Gas bubbles were extracted from the data by 3D median filtering using an 8 × 8 × 8 voxel cubic kernel, then hysteresis thresholding, using the Fiji image analysis software (Schindelin et al., 2012). Aerenchymatous roots were extracted using a region‐growth method (Keyes et al., 2013) followed by manual analysis of remaining roots in Avizo 9.0.0. The gas bubble geometry was subtracted from the root geometry to remove coclassified voxels. The spatial distributions of roots and gas were classified with respect to pot depth and radial distance from a vertical axis through the centre of the plants using code written in MATLAB 2018b (MathWorks, Massachusetts, USA).

We transformed the scanned root and gas data into volumetric spatial data (root length density, *L*_V_, and volumetric gas content, *θ*_G_) using the conversion that one voxel edge length was equivalent to 0.04 mm. Each scan was 5.8 cm (1,450 pixels) in depth, with approximately 5 cm of soil above the upper edge and 6 cm below the lower edge. The *L*_V_ and *θ*_G_ data were extrapolated over the entire depth by fitting three‐dimensional Gaussian distributions to the pooled data for the three replicates for each planting density:

(7) $$X\left(r,z\right)=A\exp\left[-\frac{{\left\{\left(r-r_{0}\right)\cos\varphi +\left(z-z_{0}\right)\sin\varphi \right\}}^{2}}{2{\sigma_{r}}^{2}}-\frac{{\left\{\left(r-r_{0}\right)\sin\varphi +\left(z-z_{0}\right)\cos\varphi \right\}}^{2}}{2{\sigma_{z}}^{2}}\right],$$

where *X* is either *L*_V_ or *θ*_G_ and *φ*, *σ*_*r*_, and *σ*_*z*_ are the corresponding fitting coefficients. Parameters were fitted in MATLAB using the *fmincon* function to minimize the square difference between the measurements and Equation (7).

### 2.4 Model parameterization

We solved Equation (1) for each of the three gases CO_2_, CH_4_, and N_2_ subject to the stated boundary conditions and Equations (2)–(6) using standard numerical methods. We parameterized the model as follows.

First, we used preset values of the following parameters: (a) the three‐dimensional distribution of *L*_V_ obtained from the root images as described in the previous section; (b) *K*_*θ*_, $S_{{\mathrm{CO}}_{2},0}$, and $\alpha_{{\mathrm{CH}}_{4}}$ in Equations (2)–(4) by running the model with no roots (i.e., no rhizodeposition and no gas venting through the roots) to fit the observed concentration of dissolved CO_2_ and pH in the unplanted bulk soil and the ratio of CO_2_ to CH_4_ measured in entrapped gases displaced from the soil; and (c) all other variables, except *k*_E_, *k*_T_, and *k*_V_, based on the experimental data and standard values for the constants and coefficients (Tables 1 and S1, Supporting Information).

We then fitted values of *k*_E_, *k*_T_, and *k*_V_ by running the model to obtain the best agreement between our observed and predicted three‐dimensional profiles of *θ*_G_ for each planting density, using the MATLAB *fmincon* function. A unique set of *k*_E_, *k*_T_, and *k*_V_ values was found for the whole data set by minimizing the average of the fitting errors calculated for the individual replicate runs.

The rate of generation of CO_2_ in the soil per unit soil surface was calculated from

(8) $$J_{S}=S_{{\mathrm{CO}}_{2},0}L+k_{V}\frac{2}{R^{2}}\int_{0}^{L}\int_{0}^{R}L_{V}\cdot r\;dr\;dz,$$

where *L* and *R* are the depth and radius of the soil volume, respectively. The flux through the roots was calculated from

(9) $$J_{R}=k_{T}D_{G,{\mathrm{CO}}_{2}}\frac{2}{R^{2}}\int_{0}^{L}\int_{0}^{R}L_{V}\left(C_{G,{\mathrm{CO}}_{2}}-C_{G,{\mathrm{CO}}_{2},0}\right)\cdot r\;dr\;dz.$$

The flux from the soil surface by ebullition was calculated from

(10) $$J_{E}=k_{E}\frac{2}{R^{2}}\int_{0}^{L}\int_{0}^{R}\theta_{G}C_{G,{\mathrm{CO}}_{2}}\cdot r\;dr\;dz.$$

The flux from the soil surface by diffusion was calculated from

(11) $$J_{D}=J_{S}-J_{R}-J_{E}.$$

Copies of the experimental data and the source code for the model written in FORTRAN are available from https://doi.org/10.17862/cranfield.rd.7628870.

## 3 RESULTS

### 3.1 Model fits

**Figure 2:**
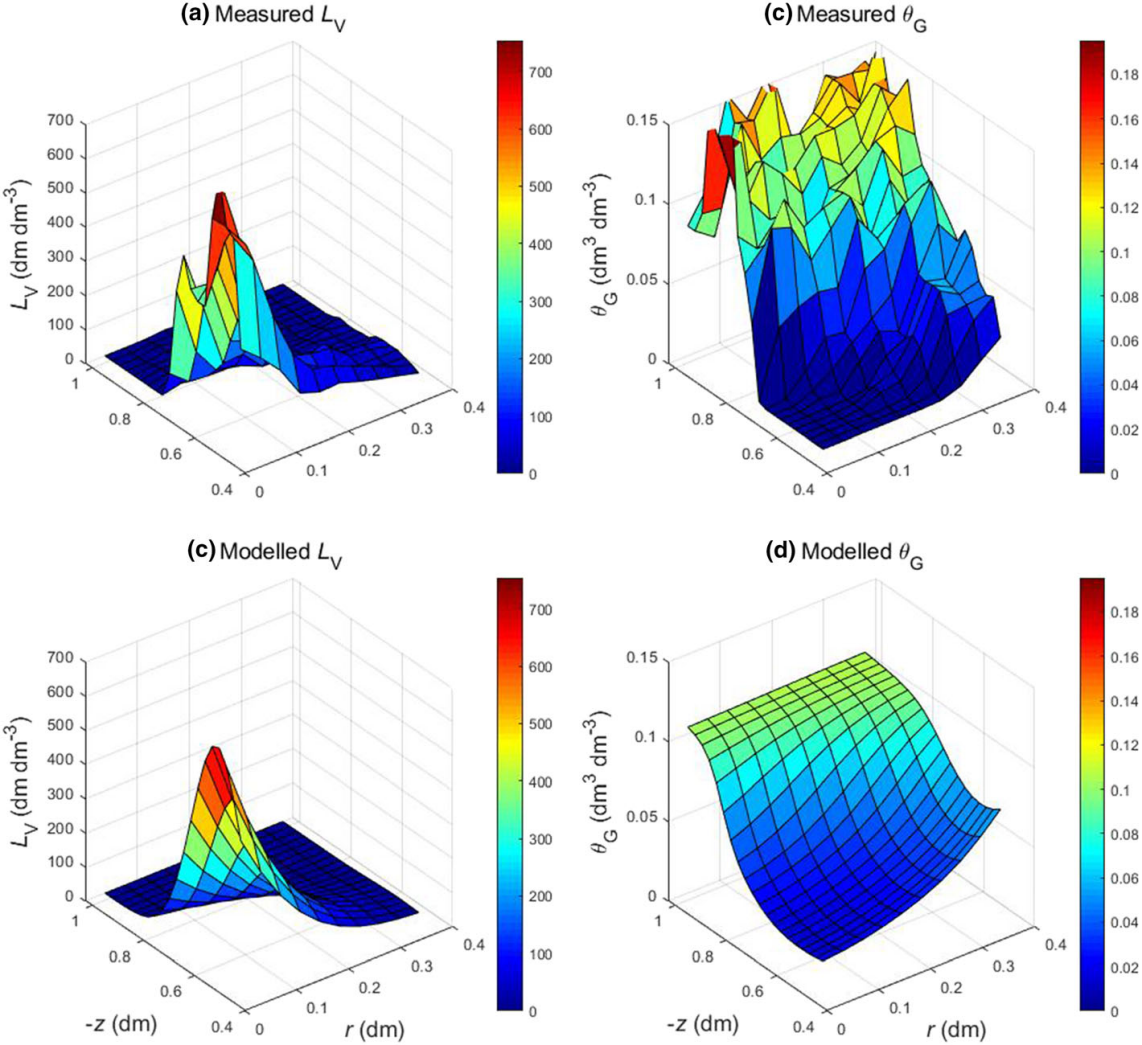
Measured and modelled results. (a, c) Root length density (*L*_V_) and (b, d) volumetric soil gas content (*θ*_G_). The measured data are for a single replicate with four plants per pot. The modelled *L*_V_ data are fits to a bimodal Gaussian distribution (Equation 7); the modelled Θ_G_ data are fits of the gas formation and transport model (Equations 1–6). Depth, *z*, is depth below floodwater–soil boundary; radius, *r*, is radial distance from the vertical axis through the middle of the plants [Colour figure can be viewed at http://wileyonlinelibrary.com]

**Figure 3:**
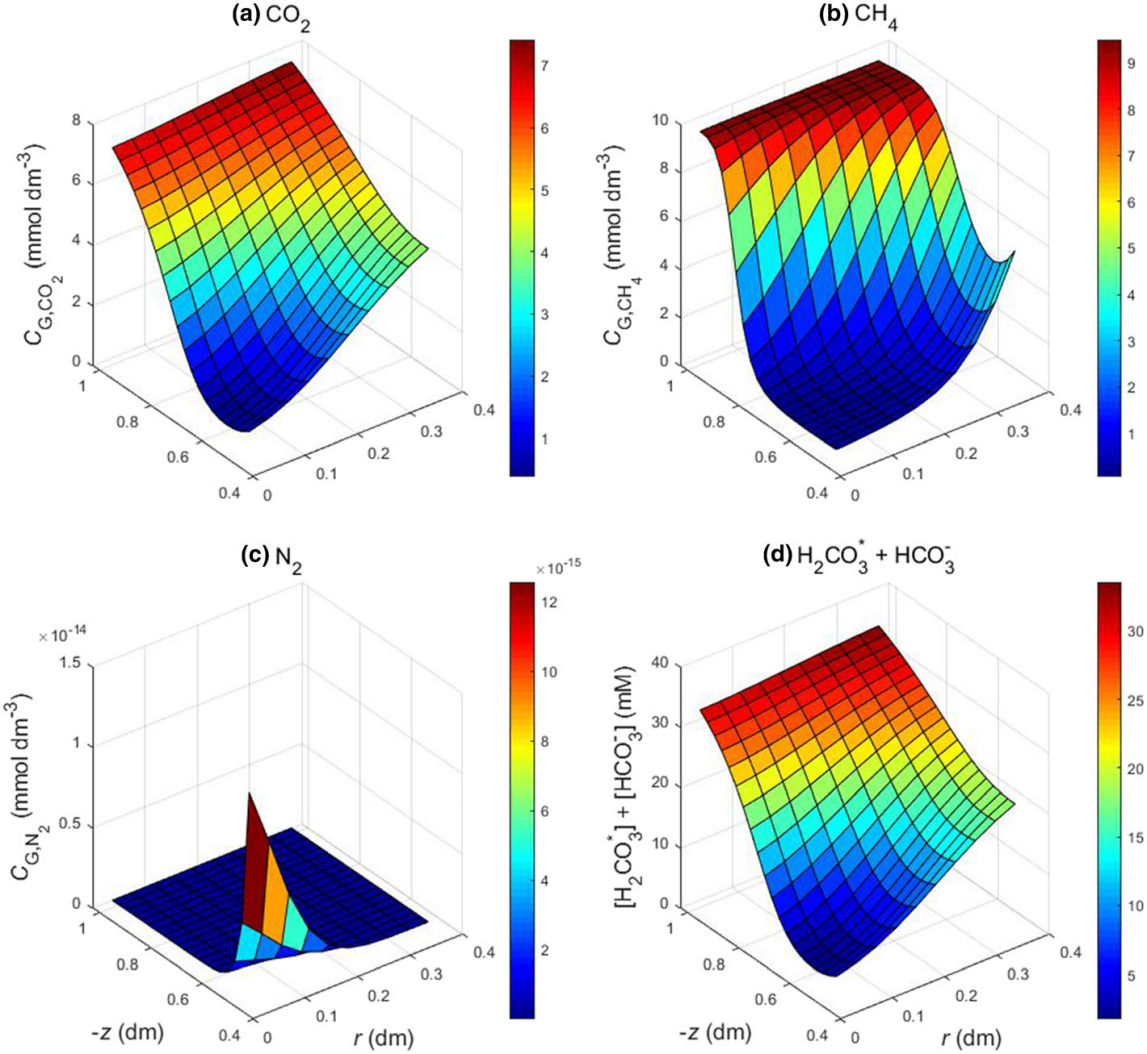
Modelled distributions of gases in the soil. (a–c) Concentrations of CO_2_, CH_4_, and N_2_ gases in soil air. (d) Concentrations of dissolved CO_2_ ([H_2_CO_3_^*^] = [CO_2_] + [H_2_CO_3_]) + HCO_3_^−^ ([HCO_3_^−^] = *K*_1_[H_2_CO_3_^*^]/[H^+^] where *K*_1_ = apparent first dissociation constant of H_2_CO_3_) in soil solution. Parameter values and root distribution as in Figure 2 [Colour figure can be viewed at http://wileyonlinelibrary.com]

Figure 2 and Figures S1 and S2 in the Supporting Information give the measured and modelled results for four plants per pot, and Figures S3–S5 in the Supporting Information give the results for one plant per pot. Figure 3 gives the calculated profiles of the different gases through the soil from the model runs in Figure 2. The fitted *k*_V_, *k*_E_, and *k*_T_ values (Table 1) are realistic (Section 4.1). So, given experimental errors, the good agreement between the observed and predicted results for both planting densities and all replicates suggests that all the important processes have been satisfactorily allowed for.

### 3.2 Sensitivity analysis

**Figure 4:**
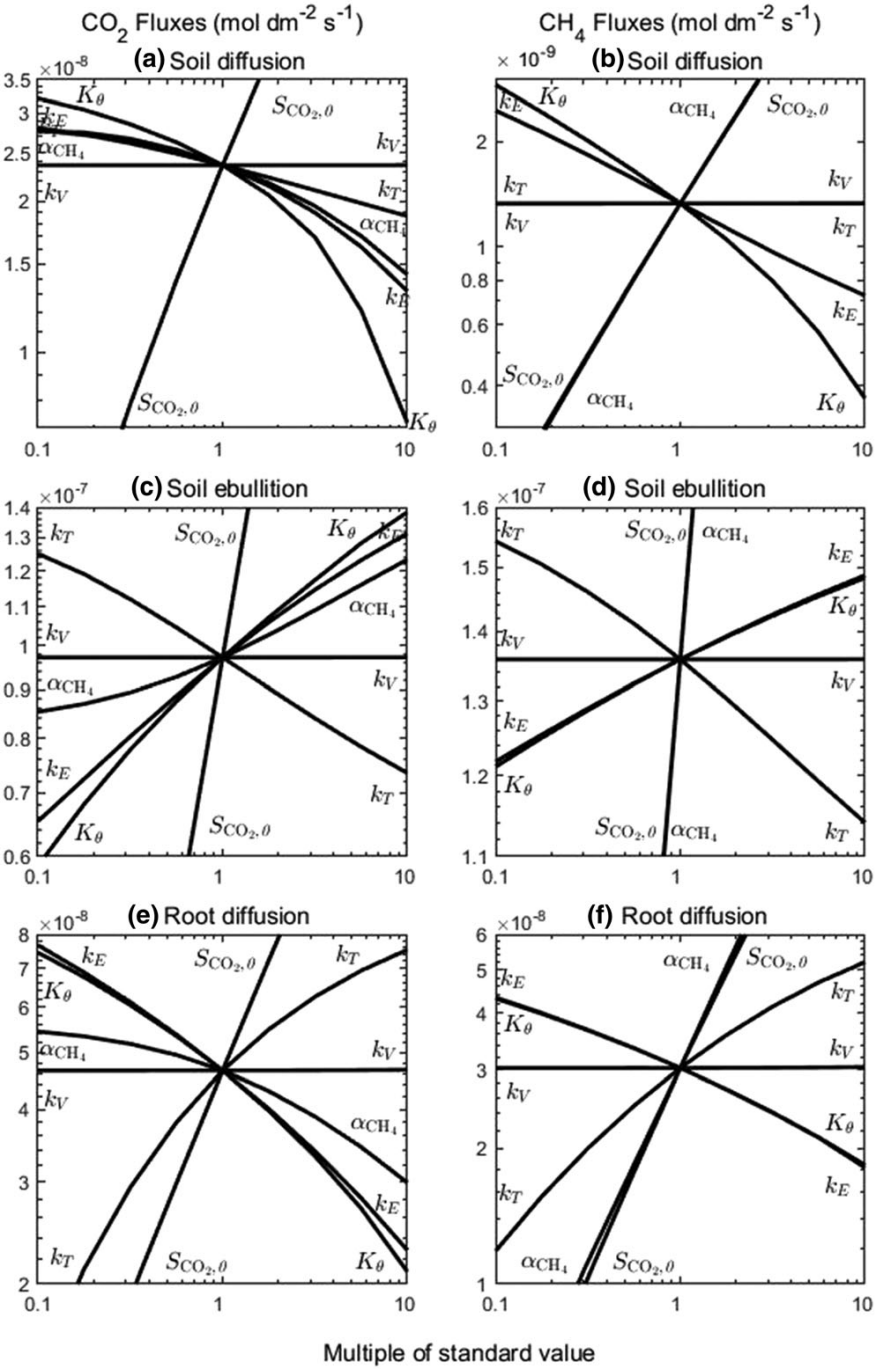
Sensitivity of model to root gas transmissivity (*k*_T_), ebullition rate constant (*k*_E_), constant for decomposition of root‐derived carbon (*k*_V_), initial soil CO_2_ production (*S*_CO2,0_), ratio of CH_4_ to CO_2_ production (*α*_CH4_), and *K*_θ_ in Equation (2). Other parameters as for Figures 2 and 3 (Table 1)

Figure 4 shows the sensitivity of the model to its input parameters. For the standard parameter values, the rate of CO_2_ production from soil carbon per pot (= $S_{{\mathrm{CO}}_{2},0}$ × soil volume) = 0.016 mol day^−1^ and the flux of CO_2_ through the roots per pot (=*J*_R_ × soil surface area) = 0.005 mol day^−1^. The plant shoot growth over 28 days was 2.2 ± 0.4 g dry weight per pot ≈ 0.073 mol C. Assuming exponential growth and equal root and shoot growth, this is equivalent to approximately 0.015 mol day^−1^ after 28 days. So the CO_2_ flux though the roots was approximately a third of the daily rate of photosynthesis. For the standard values, the proportions of total CO_2_ escaping though the roots and by diffusion and ebullition from the soil surface are 28%, 14%, and 58%, respectively, and the proportions of CH_4_ escaping via these pathways are 18%, 1%, and 81%, respectively.

Over the hundredfold range in values shown in Figure 4, the fluxes through all three routes are most sensitive to the soil carbon‐derived respiration, $S_{{\mathrm{CO}}_{2},0}$. The fluxes are also sensitive to the ebullition rate constant, *k*_E_, and the constant, *K*_θ_, in Equation (2). However, these are fitting parameters for the soil and are themselves sensitive to the value of $S_{{\mathrm{CO}}_{2},0}$, a large *E*_i_ following from a large *S*_i_ in Equation (1); so they are less relevant to our main theme of venting through the roots. The constant for root carbon‐derived respiration, *k*_V_, is unimportant at the high $S_{{\mathrm{CO}}_{2},0}$ value of our humose experimental soil; it will be more important at lower $S_{{\mathrm{CO}}_{2},0}$ values. The CO_2_ and CH_4_ fluxes are also sensitive to the ratio of CH_4_ to CO_2_ production, $\alpha_{{\mathrm{CH}}_{4}}$, and the root gas transmissivity, *k*_T_.

## 4 DISCUSSION

### 4.1 Parameter values

Wide ranges in $S_{{\mathrm{CO}}_{2},0}$ and *k*_V_ values are expected. The ricefield carbon economy—and hence $S_{{\mathrm{CO}}_{2},0}$—depends on the soil's initial organic matter content and on management of crop residues and organic manures (Greenland, 1997). Common practice is to remove part of the straw during the harvest and to burn the straw produced after threshing (Fairhurst, Witt, Buresh, & Dobermann, 2007; Greenland, 1997). The stubbles and roots are incorporated into the soil during land preparation for the following crop, and they decompose over the course of the crop. Inputs of carbon from roots—and hence *k*_V_—are as soluble exudates, insoluble secretions, and detrital root material and are also highly variable. They depend on growth conditions, healthy plants tending to be less leaky (Rose et al., 2013; van der Gon et al., 2002), and on genotype, modern rice varieties bred for high grain yield having leaner and less leaky roots than traditional varieties (Jiang et al., 2017; Maurer, Kiese, Kreuzwieser, & Rennenberg, 2018; van der Gon et al., 2002).

The ratio of CH_4_ to CO_2_ production, $\alpha_{{\mathrm{CH}}_{4}}$, depends on (a) the presence of inorganic oxidants and (b) the stochiometry of methanogenic soil organic matter decomposition and the resulting proportions of CH_4_ produced from dispoportionation of acetate versus reduction of CO_2_ with H_2_ (Reactions 2–4, Figure 1; Yao & Conrad, 2000). In general, the former dominates (Yao & Conrad, 2000), and $\alpha_{{\mathrm{CH}}_{4}}$ = 1 is typical (Kirk, 2004). A large proportion of the CH_4_ flux will be oxidized to CO_2_ by methanotrophic bacteria in the rhizosphere and oxic floodwater–soil interface; up to 95% of the root‐mediated CH_4_ flux is oxidized to CO_2_ (Arah & Kirk, 2000; Cho, Schroth, & Zeyer, 2012; Hernández, Dumont, Yuan, & Conrad, 2015; Reid, Pal, & Jaffe, 2015; van Bodegom, Stams, Mollema, Boeje, & Leffelaar, 2001). The net root CO_2_ flux will be correspondingly greater.

The root gas transmissivity, *k*_T_, depends on such variables as aerenchyma volume fraction, the permeability of root tips and laterals, root architecture, and growth stage (Kirk, 2003; Yamauchi, Colmer, Pederson, & Nakazono, 2018). The value of *k*_T_ will also influence the degree of aerobic CO_2_ generation and CH_4_ oxidation in the rhizosphere. Other things being equal, a high *k*_T_ value reduces rather than enhances net CH_4_ emission because it allows increased oxygenation of the rhizosphere (Arah & Kirk, 2000; Jiang et al., 2017). There is not much published information with which to judge our *k*_T_ values directly. However, from the wealth of information on the root pathway for CH_4_ emissions from rice, our root fluxes of CO_2_ are highly plausible.

### 4.2 Mechanisms of CO_2_ entry into the root

To reach the aerenchyma in the root cortex, dissolved CO_2_ and HCO_3_^−^ in the soil solution must pass through the root wall and epidermal tissues. Under anoxic conditions in submerged soil, the rice root system develops a layer of suberized cells in the walls of primary roots starting 1–1.5 cm behind the root tip (Yamauchi et al., 2018). This layer is highly impermeable to O_2_—and by implication to CO_2_—and so restricts radial loss of O_2_ to the soil and thereby allows a greater length of root to be aerated (Yamauchi et al., 2018). The rice root system typically comprises coarse, aerenchmymatous, primary roots with gas‐impermeable walls conducting O_2_ to short, fine, gas‐permeable laterals, which have a much greater surface area per unit mass than the primary roots. Kirk (2003) shows that this architecture provides the greatest absorbing surface for nutrients per unit aerated root mass. The same argument would apply to the absorption of CO_2_ by the root system. A further pathway for soil CO_2_ into the aerenchyma may be via the basal stem tissue at the root–shoot junction below the soil surface (Pedersen, Pulido, Rich, & Colmer, 2011).

After crossing the root wall, the dissolved CO_2_ in the root apoplast must pass through the epidermal tissue. The passive apoplastic route through the epidermis is obstructed by the Casparian strip and so CO_2_ or HCO_3_^−^ or both must cross the plasma membrane into the symplasm. Whereas uncharged CO_2_ molecules can pass through cell walls passively, HCO_3_^−^ anions cannot. This is problematic because there are no known membrane transporters for HCO_3_^−^ in higher land plants (Bloemen, McGuire, Aubrey, Teskey, & Steppe, 2013; Poschenrieder et al., 2018; Shimono, Kondo, & Evans, 2019). A boron transporter, BOR1, is reported to be homologous to an animal HCO_3_^−^ transporter (Takano et al., 2002), but there is as yet no evidence that it functions as such in plants. This implies that HCO_3_^−^ must be converted into CO_2_, which then diffuses to the cortex via the symplasm.

At the pH of the soil bulk in our experiment (7.0), 82% of the dissolved CO_2_ (H_2_CO_3_^*^ plus HCO_3_^−^) is in the form of HCO_3_^−^. Removal of CO_2_ from the soil close to root surfaces will tend to raise the soil pH (Section 4.5). But the root apoplast is generally acidified to some extent: Felle (2001) gives values below pH 6. At pH 6.5, the proportions of dissolved CO_2_ and HCO_3_^−^ are nearly equal, so the apoplastic–symplastic route will be greatly enhanced to the extent that the apoplast is acidified. We know of no studies of root apoplastic pH in rice. But given that, in general, the main form of N taken up in paddy soils is NH_4_^+^, so that cation uptake exceeds anion uptake, the apoplast is likely to be acidified. Geilfus (2017) reviews methods for measuring apoplastic pH.

The uncatalysed CO_2_ hydration–dehydration reactions, by which H_2_CO_3_ and hence HCO_3_^−^ equilibrates with CO_2_ (HCO_3_^−^ + H^+^ = H_2_CO_3_ = CO_2_ + H_2_O), are slow, and so may be rate limiting for the apolastic–symplastic pathway or degassing of CO_2_ into the aerenchyma or both. The presence of carbonic anhydrase (CA), which catalyses the reactions, in the apoplast is therefore an important question. Cytosolic CA is ubiquitous in plant tissues (DiMario, Clayton, Mukherjee, Ludwig, & Moroney, 2017), but its presence in the apoplast is less certain (Savchenko, Wiese, Neimanis, Hedrich, & Heber, 2000).

### 4.3 Fate of the CO_2_ in the root

Is the concentration of CO_2_ and associated HCO_3_^−^ in the roots sufficient to be toxic? The soil CO_2_ concentration in our experiment was equivalent to $P_{{\mathrm{CO}}_{2}}$ ≈ 20 kPa in the soil bulk but tenfold less than this at the root surface as a result of venting through the roots. Plant species well adapted to high $P_{{\mathrm{CO}}_{2}}$ in the root zone, such as rice, can thrive at $P_{{\mathrm{CO}}_{2}}$ values well above 20 kPa through mechanisms that are not well understood (Greenway et al., 2006). If the cytoplasm was in equilibrium with $P_{{\mathrm{CO}}_{2}}$ = 20 kPa and the pH was maintained at the typical value of 7.5 through the biochemical and biophysical pH stats, then the cytoplasmic HCO_3_^−^ concentration would be approximately 90 mM, which is above values at which metabolism is impaired (of the order of 50 mM or possibly as low as 10 mM for some enzyme systems—Greenway et al., 2006), whereas at $P_{{\mathrm{CO}}_{2}}$ = 2 kPa, as calculated for the soil at the root surface, the HCO_3_^−^ concentration would be only about 9 mM, which is in the normal range (2–20 mM) and well below toxic levels. This indicates that the rate of CO_2_ venting through the roots would be sufficient to avoid toxic concentrations in root cells.

In fact, the enhanced availability of CO_2_ in the roots may have a growth stimulating effect in rice by facilitating anaplerotic production of organic acids for amino acid synthesis (Balkos, Britto, & Kronzucker, 2010; Britto & Kronzucker, 2005). In general, the main form of N taken up by rice in submerged soils is NH_4_^+^, and virtually all the NH_4_^+^ is assimilated into amino acids in the roots before being transported to the shoots (Kronzucker, Siddiqi, Glass, & Kirk, 1999). This occurs via glutamine synthetase (GS), which catalyses the incorporation of NH_4_^+^ into the organic pool, and phosphoenolpyruvate carboxylase (PEPC), which fixes CO_2_ into oxaloacetate and malate so providing carbon skeletons for the GS pathway. In principle, if other factors are nonlimiting, increased CO_2_ supply in the roots would allow greater N assimilation.

The PEPC pathway might be a significant sink for root CO_2_. An upper estimate of the size of this sink can be got from the rate of N uptake by the roots with the crude assumption that all the N is taken up as NH_4_^+^ and assimilated via GS and PEPC. From the plant growth rate (0.45 g day^−1^ at 28 days after transplanting—Section 3.2) and N content (approximately 15 mg g^−1^—Affholder et al., 2017), the rate of N uptake was approximately 0.48 mmol day^−1^, which is less than 10% of the CO_2_ flux through the roots. In fact, a significant part of N uptake by rice in submerged soils is as NO_3_^−^, formed by nitrification of NH_4_^+^ in the rhizosphere (Kirk & Kronzucker, 2005), and most of the NO_3_^−^ will be assimilated in the shoots rather than the roots (Kronzucker et al., 1999). We conclude the flux of CO_2_ through PEPC in the roots will be small compared with the net CO_2_ flux. This is consistent with the assumption implicit in the model that, at steady state, effectively all the CO_2_ entering the roots diffuses to the shoots via the aerenchyma (Equation 6).

### 4.4 Fate of the CO_2_ reaching the shoot

Could recycling of root‐ and soil‐derived CO_2_ through the roots to the shoots provide a source of CO_2_ for photosynthesis? The soil‐derived CO_2_ flux through the plants was equivalent to approximately a third of the daily rate of photosynthesis, that is, 20% of the actual rate of photosynthesis given that the photoperiod was 13.5 hr. This suggests a large potential source for photosynthesis. We know of no data on this point for rice plants. However, measurements with emergent wetland plants such as Phragmites suggest sediment‐derived CO_2_ accounts for less than 1% of the carbon fixed by the shoots (Brix, 1990; Constable & Longstreth, 1994; Singer, Eshel, Agami, & Beer, 1994). Although aerenchyma provides a continuous gas pathway between the roots and leaves, the stems of rice plants contain lenticels that allow gas exchange with the atmosphere in the lower part of the canopy (Yamauchi et al., 2018). So the bulk of the root‐borne CO_2_ probably escapes from the aerenchyma before reaching the main photosynthetic tissue.

### 4.5 Other implications

Removal of soil CO_2_ through the roots has important implications for the chemistry of the rhizosphere. Removal of dissolved CO_2_ and hence H_2_CO_3_ will tend to increase the rhizosphere pH. The maximum depletion of H_2_CO_3_ + HCO_3_^−^ by the roots (Figure 3) was 30 mM, that is, 21 mmol kg^−1^, allowing for the soil water content and bulk density. Hence, from the pH buffer power of the soil (*b*_HS_ = 29 mmol·kg^−1^·pH^−1^, Section 2.2) the expected pH increase close to the roots is 0.7 units, that is, from 7.0 to 7.7. Such a pH change would substantially alter the solubility and hence plant availability of nutrients and toxicants (Kirk, 2004). For example, a pH increase in this range would make soil organic ligands more soluble and thereby solubilize soil Zn (Affholder et al., 2017). In “iron toxic” rice soils, where large concentrations of dissolved ferrous iron can severely damage the plants (Becker & Asch, 2005), H^+^ consumption in CO_2_ venting could moderate the acidification of the rhizosphere caused by ferrous iron oxidation (4Fe^2+^ + O_2_ + 10H_2_O = 4Fe(OH)_3_ + 8H^+^) and so limit the impairment of cation uptake caused by acidification (Begg et al., 1994).

The likely importance of CA in facilitating CO_2_ entry into the root and aerenchyma (Section 4.2) raises a possible link to the plant Zn nutrition. The active centre in all known plant CAs contains Zn (DiMario et al., 2017), and Zn‐deficient plants can have impaired CA activity (Sasaki, Hirose, Watanabe, & Ohsughi, 1998). Consistent with this, Affholder et al. (2017) found less CO_2_ venting through a rice genotype sensitive to soil Zn deficiency compared with a tolerant genotype.

What factors could be manipulated by plant breeding or crop management to influence soil CO_2_ uptake by rice roots? The extent of aerenchyma development and gas barriers in the root wall will be important, both for CO_2_ transmission and for oxidation of CH_4_ to CO_2_ in the rhizosphere; there are differences in both of these between rice genotypes (Yamauchi et al., 2018). There are also genotype differences in CA expression in rice (Xu, Zhang, Guan, Takano, & Liu, 2007).

## 5 CONCLUSIONS

1. Venting through the roots of CO_2_ formed in root and soil respiration is an important control on root and soil CO_2_ concentrations in submerged wetland soils over a wide range of plant and soil conditions.
2. We measured rates of CO_2_ uptake by roots equivalent to a third of the daily CO_2_ fixation in photosynthesis. Without this venting through the roots, the concentrations of CO_2_ and associated HCO_3_^−^ in root cells would have been well above levels known to be toxic to roots.
3. The removal of CO_2_ and hence H_2_CO_3_ from the soil was sufficient to increase the rhizosphere pH close to the roots by 0.7 units. That is sufficient to solubilize or immobilize various nutrients and toxicants and potentially provides an explanation for genotype differences in tolerance of nutrient deficiencies and mineral toxicities.
4. The image‐based mathematical modelling method that we used, linked to non‐invasive X‐ray CT imaging, is a powerful way of studying below‐ground plant–soil interactions.

## Supporting information


**Table S1** Values of diffusion coefficients and Henry's law constants at 25 oC (2). Also, apparent 1^st^ dissociation constant of H_2_CO_3_, K1 = 4.45 × 10^‐7^ mol dm^‐3^; saturating water pressure, $P_{H_{2}O}$ = 5 kPa; gas constant, R = 8.314 dm^3^ kPa K^‐1^ mol^‐1^
**Fig. S1** Measured and modelled results for the second replicate with 4 plants per pot.
**Fig. S2** Measured and modelled results for the third replicate with 4 plants per pot.
**Fig. S3** Measured and modelled results for the first replicate with 1 plant per pot.
**Fig. S4** Measured and modelled results for the second replicate with 1 plant per pot.
**Fig. S5** Measured and modelled results for the third replicate with 1 plant per pot.
