# Supplementary material for: Soil carbon dioxide venting through rice roots
Source: Plant Cell Environ. 2019 Aug 19;42(12):3197–207. doi: 10.1111/pce.13638 (PMC6972674; doi:10.1111/pce.13638)

**Soil carbon dioxide uptake by rice roots**

Guy J.D. Kirk, Andrea Boghi, Marie-Cecile Affholder, Samuel D. Keyes, James Heppell, and Tiina Roose

**Supporting Information**

**Table S1** Values of diffusion coefficients and Henry’s law constants at 25 ^o^C (2). Also, apparent 1^st^ dissociation constant of H_2_CO_3_, *K*_1_ = 4.45 × 10^-7^ mol dm^-3^; saturating water pressure,$P_{\text{H}_{2}\text{O}}$= 5 kPa; gas constant, *R* = 8.314 dm^3^ kPa K^-1^ mol^-1^

| Symbol | Definition | Value | | | Units |
| --- | --- | --- | --- | --- | --- |
|  |  | CO_2_ | CH_4_ | N_2_ |  |
| *D*_G_ | diffusion coefficient in air | 1.55 × 10^-3^ | 2.20 × 10^-3^ | 2.04 × 10^-3^ | dm^-2^ s^-1^ |
| *D*_L_ | diffusion coefficient in solution | 1.18 × 10^-7^ | 1.73 × 10^-7^ | 2.02 × 10^-7^ | dm^-2^ s^-1^ |
| *K*_H_ | Henry’s law constant | 8.30 × 10^-1^ | 3.15 × 10^-2^ | 1.62 × 10^-2^ | - |

**Fig. S1** Measured and modelled results for the second replicate with 4 plants per pot.


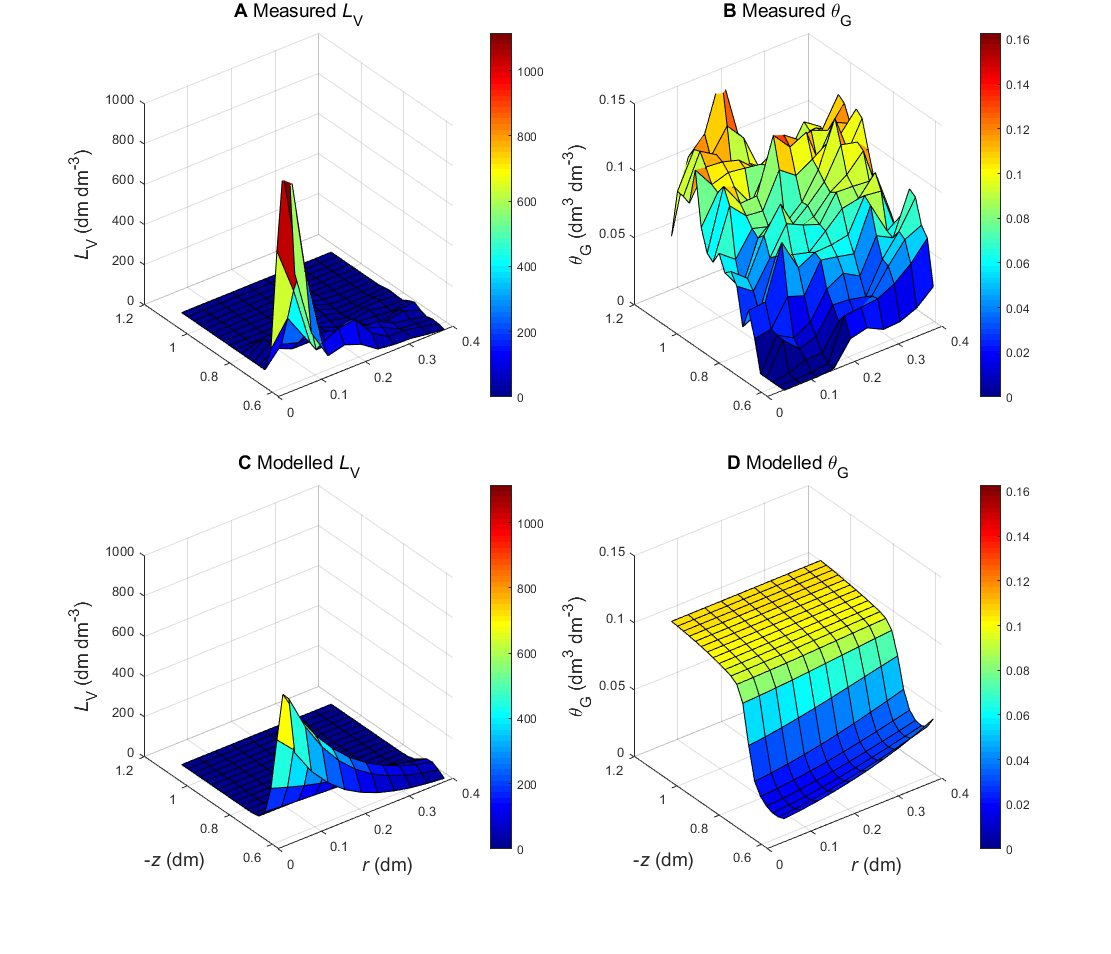


**Fig. S2** Measured and modelled results for the third replicate with 4 plants per pot.


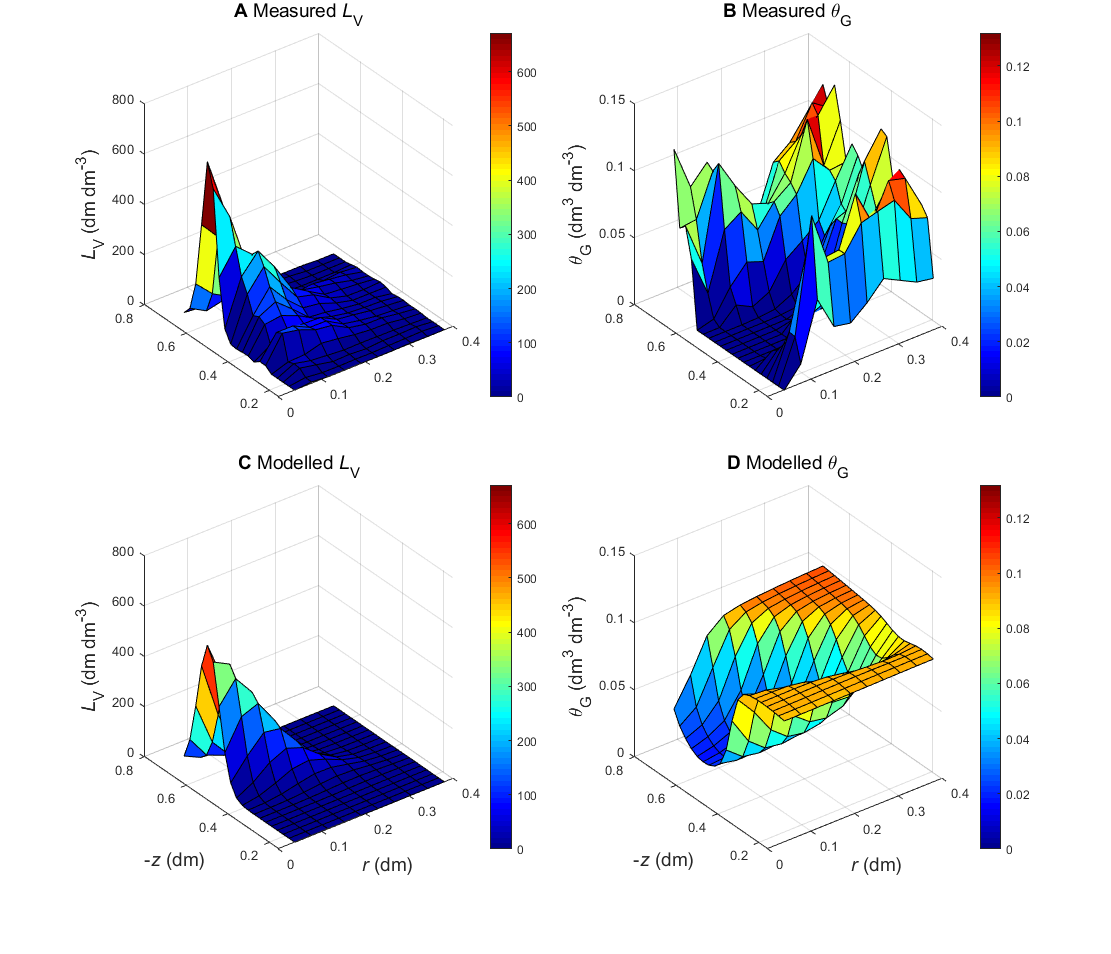


**Fig. S3** Measured and modelled results for the first replicate with 1 plant per pot.


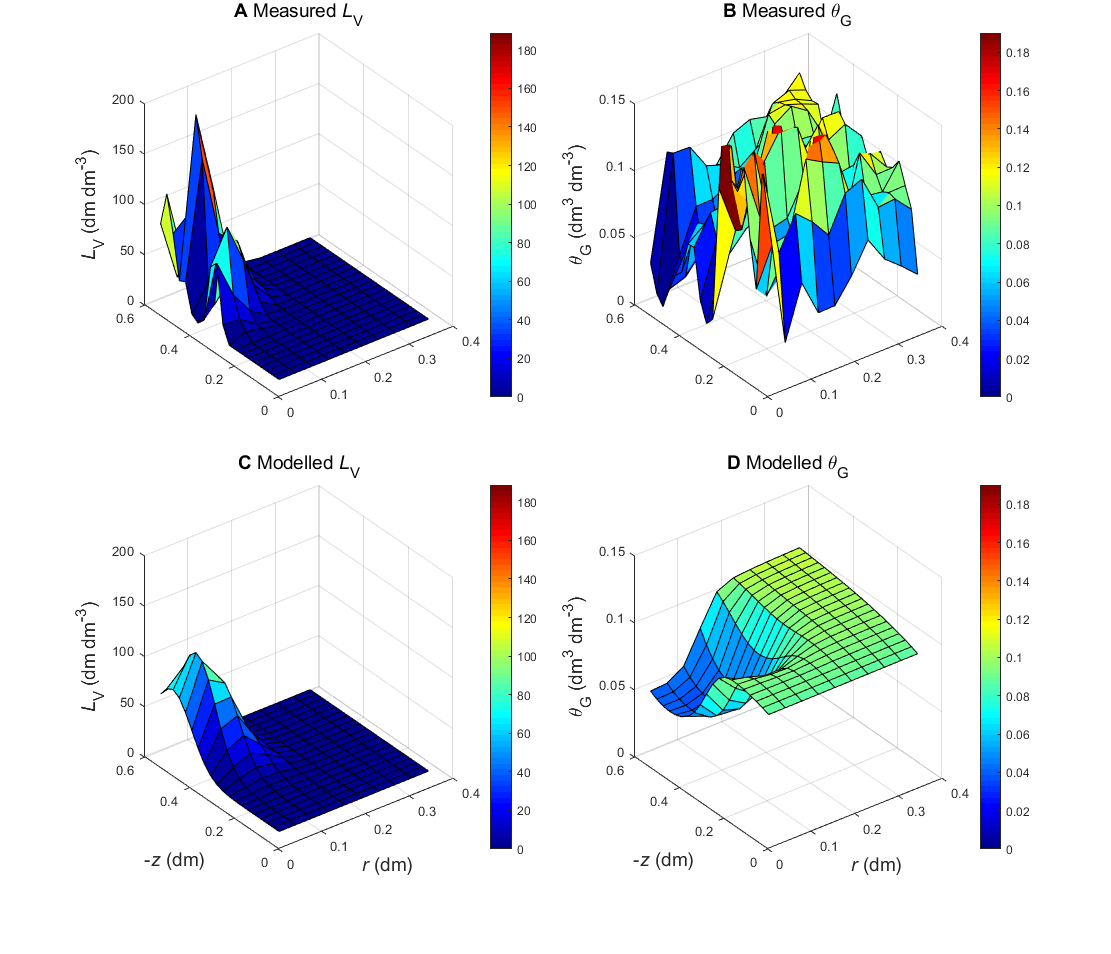


**Fig. S4** Measured and modelled results for the second replicate with 1 plant per pot.


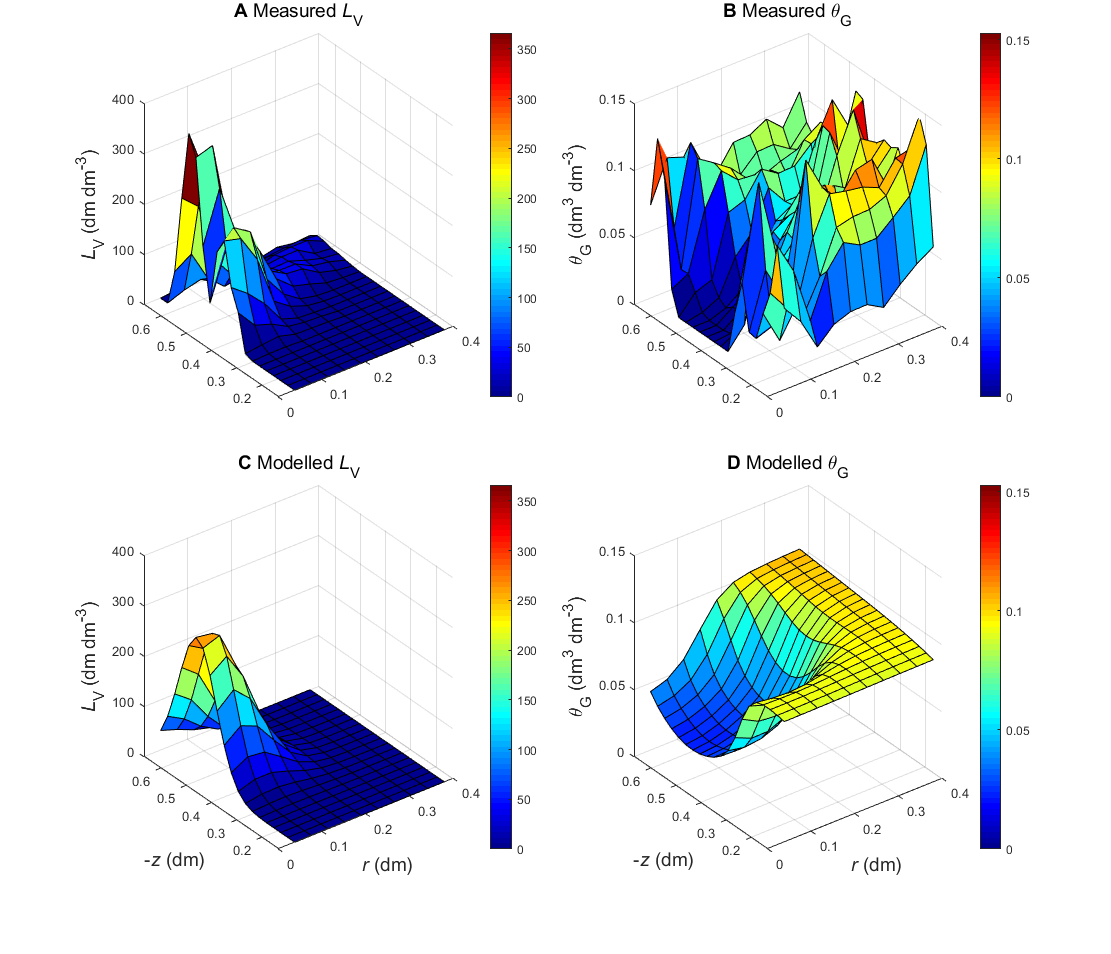


**Fig. S5** Measured and modelled results for the third replicate with 1 plant per pot.


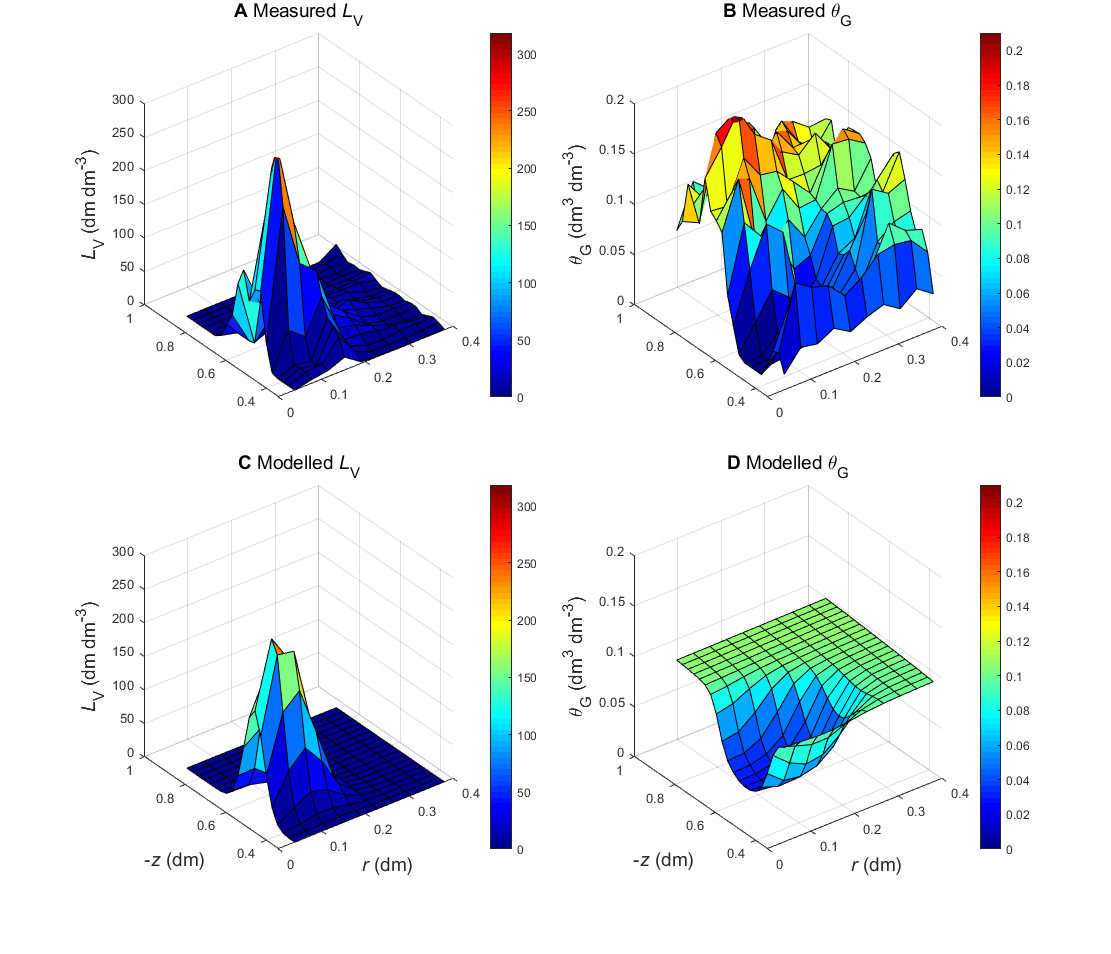

Supplement: Supplementary file 1 — Table S1 Values of diffusion coefficients and Henry's law constants at 25 oC (2). Also, apparent 1st dissociation constant of H2CO3, K1 = 4.45 × 10‐7 mol dm‐3; saturating water pressure, PH2O = 5 kPa; gas constant, R = 8.314 dm3 kPa K‐1 mol‐1 Fig. S1 Measured and modelled results for the second replicate with 4 plants per pot. Fig. S2 Measured and modelled results for the third replicate with 4 plants per pot. Fig. S3 Measured and modelled results for the first replicate with 1 plant per pot. Fig. S4 Measured and modelled results for the second replicate with 1 plant per pot. Fig. S5 Measured and modelled results for the third replicate with 1 plant per pot. [file PCE-42-3197-s001.zip › PCE13638-supp-0001-Supp Material.docx]
