# Supplementary material for: Soil carbon dioxide venting through rice roots
Source: Plant Cell Environ. 2019 Aug 19;42(12):3197–207. doi: 10.1111/pce.13638 (PMC6972674; doi:10.1111/pce.13638)

# Soil carbon dioxide uptake by rice roots

Guy J.D. Kirk, Andrea Boghi, Marie-Cecile Affholder, Samuel D. Keyes, James Heppell, and Tiina Roose

## SUPPORTING INFORMATION

**Table S1** Values of diffusion coefficients and Henry's law constants at 25 $^{o}C$ (2). Also, apparent 1st dissociation constant of $H_2CO_3$, $K_1$ = 4.45 × $10^{-7}$ mol $dm^{-3}$; saturating water pressure, $P_{H_2O}$ = 5 kPa; gas constant, $R$ = 8.314 $dm^3$ kPa $K^{-1}$ $mol^{-1}$

| Symbol | Definition | Value | | | Units |
|---|---|---|---|---|---|
| | | $CO_2$ | $CH_4$ | $N_2$ | |
| $D_G$ | diffusion coefficient in air | 1.55 × $10^{-3}$ | 2.20 × $10^{-3}$ | 2.04 × $10^{-3}$ | $dm^{-2}$ $s^{-1}$ |
| $D_L$ | diffusion coefficient in solution | 1.18 × $10^{-7}$ | 1.73 × $10^{-7}$ | 2.02 × $10^{-7}$ | $dm^{-2}$ $s^{-1}$ |
| $K_H$ | Henry's law constant | 8.30 × $10^{-1}$ | 3.15 × $10^{-2}$ | 1.62 × $10^{-2}$ | - |

**Fig. S1** Measured and modelled results for the second replicate with 4 plants per pot.

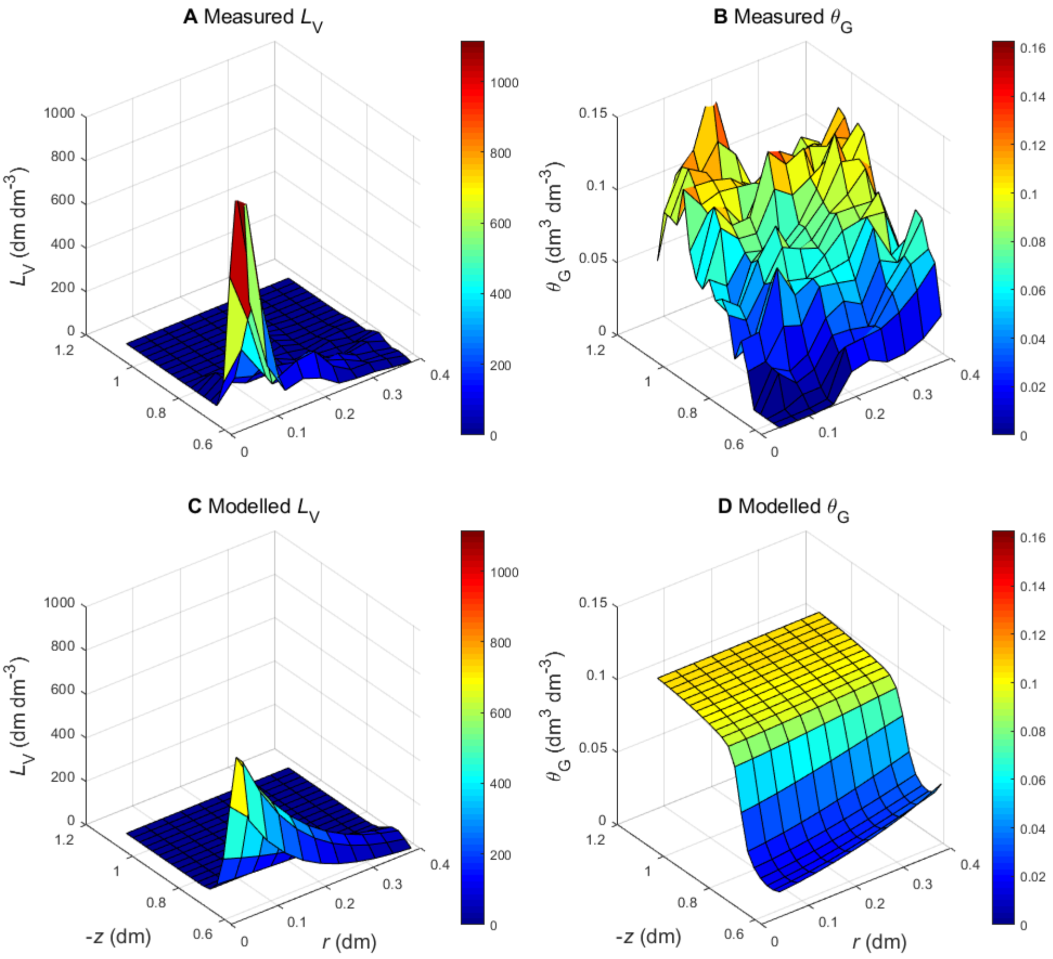

**Fig. S2** Measured and modelled results for the third replicate with 4 plants per pot.

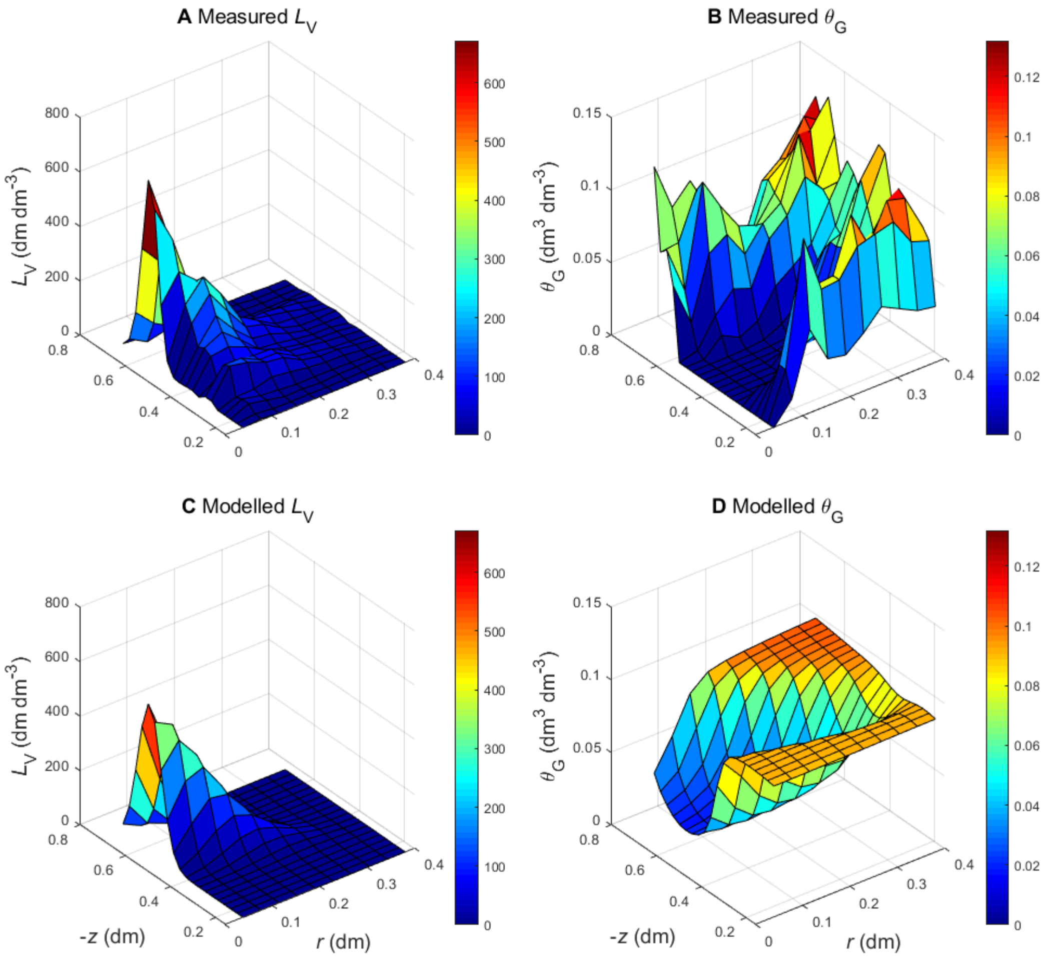

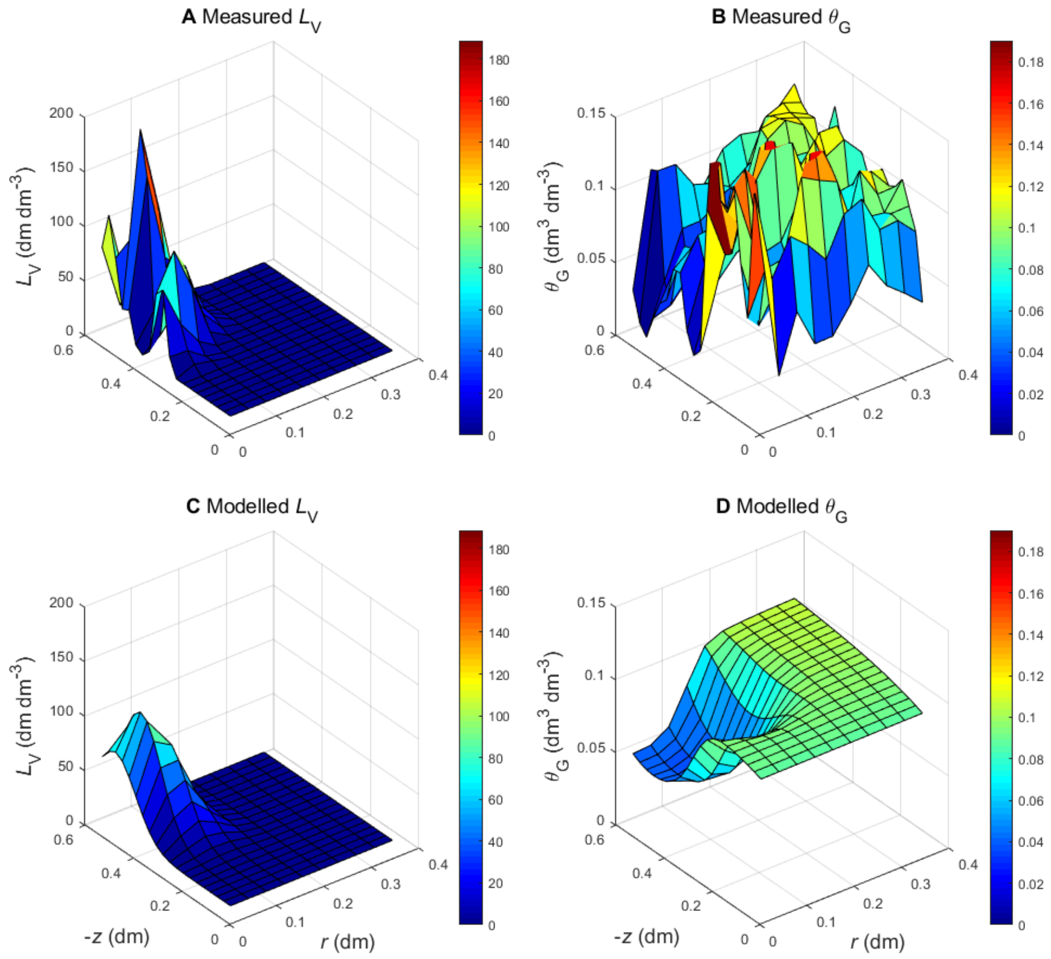


**Fig. S3** Measured and modelled results for the first replicate with 1 plant per pot.

**Fig. S4** Measured and modelled results for the second replicate with 1 plant per pot.

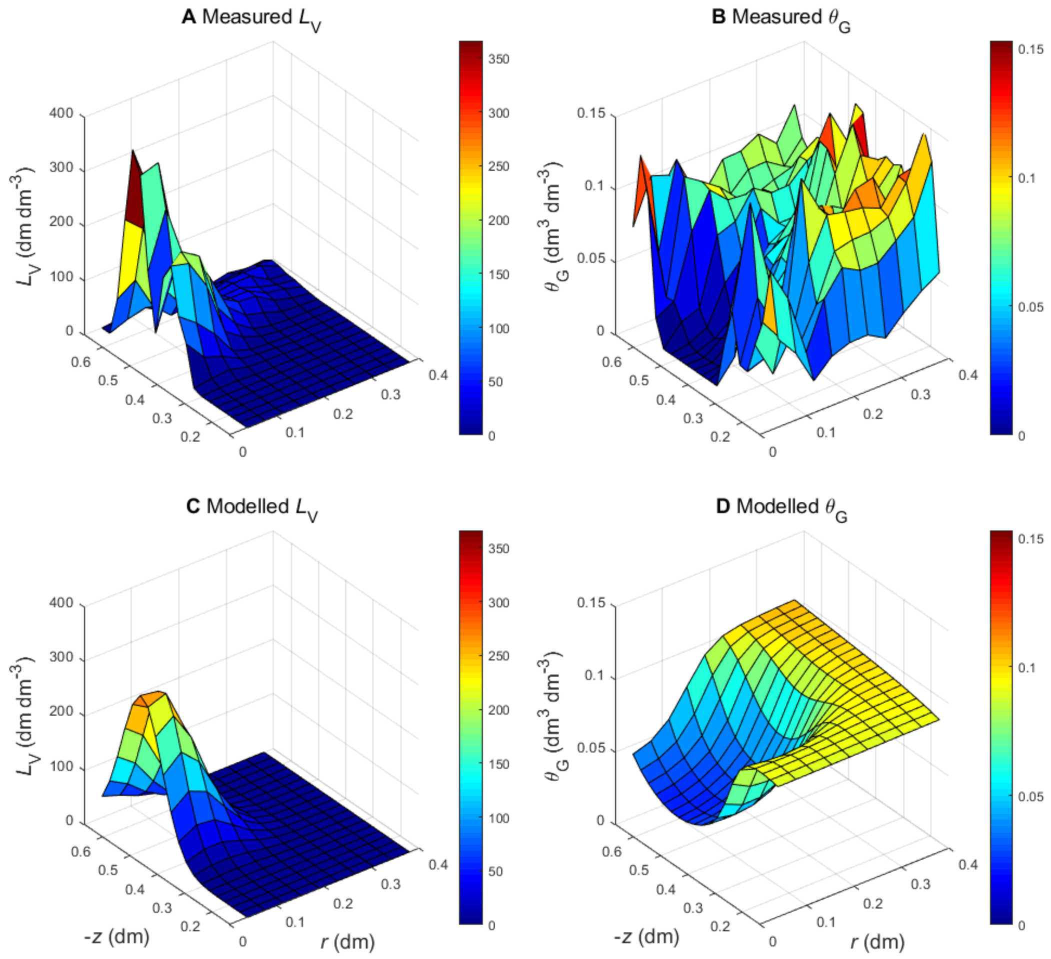

**Fig. S5** Measured and modelled results for the third replicate with 1 plant per pot.

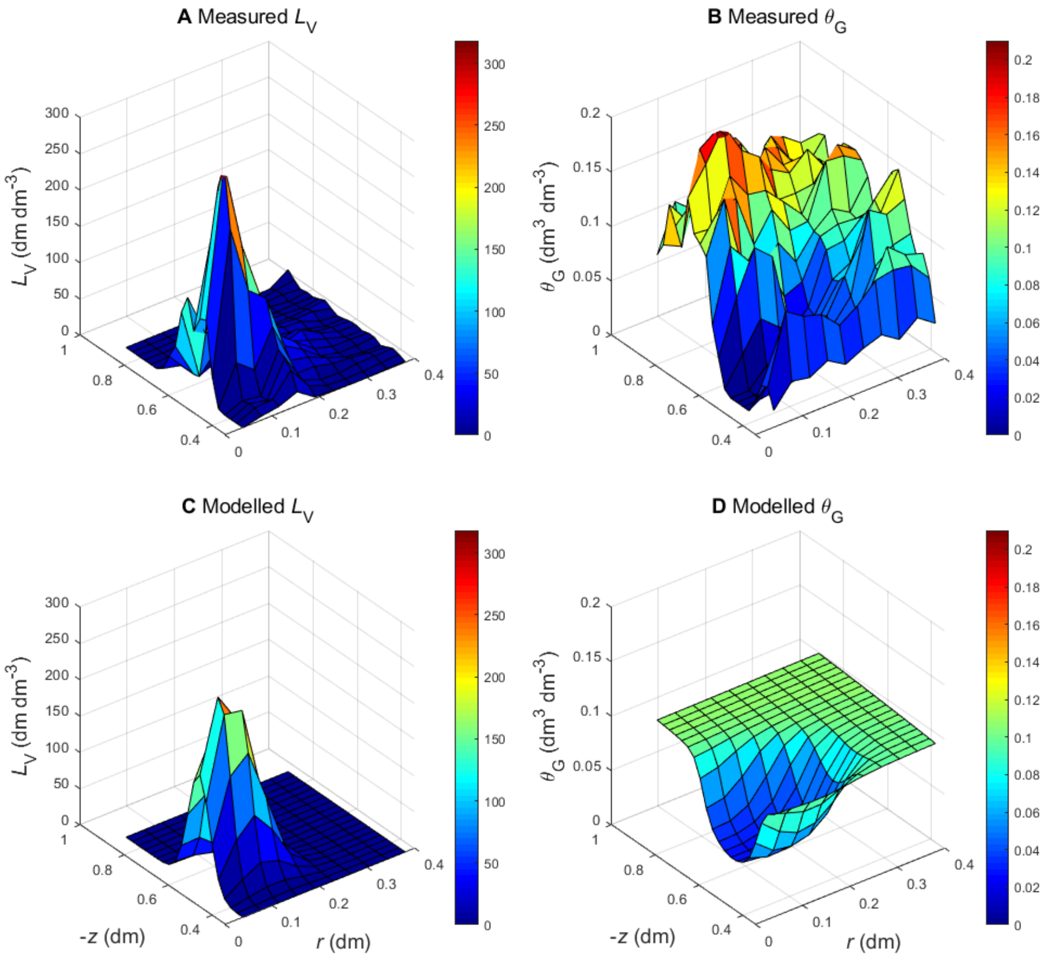

Supplement: Supplementary file 1 — Table S1 Values of diffusion coefficients and Henry's law constants at 25 oC (2). Also, apparent 1st dissociation constant of H2CO3, K1 = 4.45 × 10‐7 mol dm‐3; saturating water pressure, PH2O = 5 kPa; gas constant, R = 8.314 dm3 kPa K‐1 mol‐1 Fig. S1 Measured and modelled results for the second replicate with 4 plants per pot. Fig. S2 Measured and modelled results for the third replicate with 4 plants per pot. Fig. S3 Measured and modelled results for the first replicate with 1 plant per pot. Fig. S4 Measured and modelled results for the second replicate with 1 plant per pot. Fig. S5 Measured and modelled results for the third replicate with 1 plant per pot. [file PCE-42-3197-s001.zip › PCE13638-supp-0001-Supp Table 1 and Figure 1-5.pdf]
